# Spot On: Indocyanine Green-Soaked Fiducial Markers for Lung Nodules Prior to Thoracic Surgery

**DOI:** 10.1016/j.chpulm.2024.100131

**Published:** 2024-12-25

**Authors:** Bryan S. Benn, Hasnain Bawaadam, Elizabeth M. Colwell, Matthew D. Peterson, William B. Tisol, Abesh Niroula, Wissam S. Jaber, Onkar V. Khullar, Kelly Daymude, Chinh T. Phan, Luis A. Godoy, Devon Anderson, Michelle Lagana, Elizabeth A. Yu, Tomomi Oka, Mendy Lum, Pallav L. Shah, Ganesh Krishna

**Affiliations:** a Department of Pulmonary Medicine, Respiratory Institute, Cleveland Clinic, Cleveland, OH; b Department of Interventional Pulmonary and Critical Care Medicine, Advocate Aurora Medical Center, Kenosha, WI; c Department of Cardiothoracic Surgery, Advocate Aurora St Lukes Medical Center, Milwaukee, WI; d Division of Pulmonary, Allergy, Critical Care and Sleep Medicine, Emory University School of Medicine, Atlanta, GA; e Division of Cardiothoracic Surgery, Emory University, Atlanta, GA; f Emory Healthcare, Atlanta, GA; g Division of Pulmonary, Critical Care, and Sleep Medicine, Department of Internal Medicine, Sacramento, CA; h Division of General Thoracic Surgery, Department of Surgery, University of California, Davis Health, Sacramento, CA; i Division of Pulmonary, Critical Care, Allergy and Sleep Medicine, Department of Medicine, University of California, San Francisco, CA; j Division of Pulmonary and Critical Care Medicine, Department of Medicine, Palo Alto, CA; k Department of Cardiothoracic Surgery, Palo Alto Medical Foundation, Palo Alto, CA; l Respiratory Medicine, El Camino Hospital, Mountain View, CA; m Royal Brompton Hospital, London, England; n National Heart & Lung Institute, Imperial College, London, England

**Keywords:** dye marking, fiducial maker, indocyanine green, lung nodule, lung surgery

## Abstract

**Background:**

Peripheral pulmonary lesions (PPLs) are increasingly identified and often require a tissue diagnosis to guide treatment. Although a surgical resection may combine diagnosis and treatment, it may lead to excessive healthy tissue being removed if the lesion is difficult to localize. Bronchoscopic PPL marking before surgery facilitates this process, but it is limited by current technologies. Advances in procedural techniques may improve this process.

**Research Question:**

What is the impact of using indocyanine green-soaked fiducial markers (ICG-Fs) to mark PPLs before surgery compared with unmarked resected PPLs?

**Study Design and Methods:**

A retrospective review of patients from 4 institutions with PPLs undergoing bronchoscopy with ICG-F marking (54 nodules) before resection were compared with unmarked nodules (63 nodules). Demographic data, nodule characteristics, procedural and surgical information, and final pathology results were obtained.

**Results:**

Demographics were similar between the groups. PPLs were smaller in the ICG-F marked group (axial: ICG-F marked: 14.39 ± 5.39 vs unmarked: 20.31 ± 14.24 mm; *P* = .0036; coronal: ICG-F marked: 12.66 ± 5.13 vs unmarked: 16.43 ± 10.51 mm; *P* = .0214). All ICG-F marked lesions were visible with illumination at surgery immediately after bronchoscopy or up to 13 days later. Mean weight (58 ± 77 vs 145 ± 80 g; *P* < .001) and size (9.07 ± 6.0 × 4.73 ± 3.6 × 2.42 ± 1.23 vs 14.63 ± 6.08 × 8.70 ± 4.36 × 4.08 ± 1.94 mm; *P* < .001 for all) of the resected ICG-F specimens were significantly decreased compared with unmarked PPLs. Operative time was increased in the ICG-F marked group (165 ± 53 vs 136 ± 43 minutes; *P* = .0021).

**Interpretation:**

Our findings indicate that ICG-F is a safe and accurate procedure to facilitate lung sparing surgery of otherwise undetectable PPLs immediately after bronchoscopic placement or up to 13 days later.

**Take-Home Points**

**Study Question:** What is the impact of using indocyanine green-soaked fiducial markers (ICG-Fs) to mark peripheral pulmonary lesions (PPLs) before surgery compared with unmarked resected PPLs?

**Results:** All ICG-F marked lesions were visible with illumination at surgery immediately after bronchoscopy or up to 13 days later without any complication associated with the marking procedure or surgery.

**Interpretation:** Our results indicate that ICG-F is a safe and accurate procedure to facilitate lung sparing surgery of otherwise undetectable PPLs immediately after bronchoscopic placement or up to 13 days later.

Lung cancer remains a leading cause of mortality throughout the world,^1^ with many patients presenting with late-stage disease.^2^ Randomized controlled trials have shown that annual screening with low-dose CT scans can reduce overall mortality,^3^^,^^4^ and patients presenting at earlier stages have improved survival outcomes.^5^^,^^6^ Estimates suggest that approximately 4.8 million chest CT scans are performed yearly in the United States, with approximately 1.57 million pulmonary nodules identified.^7^

Although most of these indeterminate peripheral pulmonary lesions (PPLs) will be benign, many need a tissue diagnosis to guide treatment.^8^ Diagnosis and treatment may be combined during a surgical resection, which is safe and effective, but may lead to excision of benign disease^9^, ^10^, ^11^ and/or excessive healthy lung tissue if the PPL is difficult to localize. Although different techniques are used to improve this procedure,^12^^,^^13^ minimizing healthy lung tissue removal during surgery remains a challenge.

Studies have shown that minimally invasive lung sparing surgery is noninferior to lobectomy for patients with early stage lung cancer. Compared with lobectomy, patients undergoing sublobar resection had comparable overall survival,^14^ fewer postoperative complications,^15^ and improved postoperative measures of quality of life.^16^ Patients undergoing segmentectomy had a superior 5-year overall survival rate compared with those undergoing lobectomy.^17^ Thus, there is a growing desire to provide appropriate patients with minimally invasive lung sparing surgery.^18^

One approach to facilitate surgical wedge resection is to localize the lesion via bronchoscopy.^19^^,^^20^ Multiple techniques have been used, including pleural marking with stains and dyes and placement of fiducial markers in and around the lesion,^19^^,^^20^ depending on availability and physician preference without any clear guidelines. Bronchoscopic fiducial marker placement is safe and effective^21^ and may reduce time to surgery compared with percutaneous marking.^22^ Similar results have been seen with pleural dye marking.^23^^,^^24^

Successful nodule marking, however, lacks a clear definition because it is usually subjectively assessed by the proceduralist.^21^ Procedure failure occurs when the surgeon is unable to clearly visualize or palpate the lesion for resection, which may be due to dye dispersion into the pleural space^23^ or lack of fiducial marker retention.^25^ These errors potentially increase procedural time, change the operative approach, increase the amount of healthy lung tissue resected, and result in failure to remove the diseased portion of the lung.^23^

Nodule marking may also be limited by the accuracy of the bronchoscopic portion of the procedure. Previous studies have shown that current electromagnetic navigation-guided bronchoscopy has a limited sensitivity for diagnosing malignancy.^26^ Initial studies using shape-sensing robotic-assisted navigational bronchoscopy (ssRANB) technology have shown an improvement in procedural sensitivity for malignancy, including for lesions < 2 cm in diameter.^27^, ^28^, ^29^, ^30^, ^31^

A report has also highlighted the potential to improve the PPL marking process further by coupling dye and fiducial marking together in 1 procedure.^32^ Placement of indocyanine green-soaked fiducial markers (ICG-Fs) allowed for surgical resection to occur up to 9 days after bronchoscopy to overcome potential logistical and organizational constraints.^32^ Thus, it is possible that using ICG-Fs may play a role not only in the timing of surgical resection, but also in facilitating lung sparing surgery. We report our experience using this PPL marking technique after its introduction at 4 institutions in the United States and sought to retrospectively compare the size and weight of resected lung specimens with ICG-F marking compared with those resected without marking.

## Study Design and Methods

### Cohort

**Figure 1:**
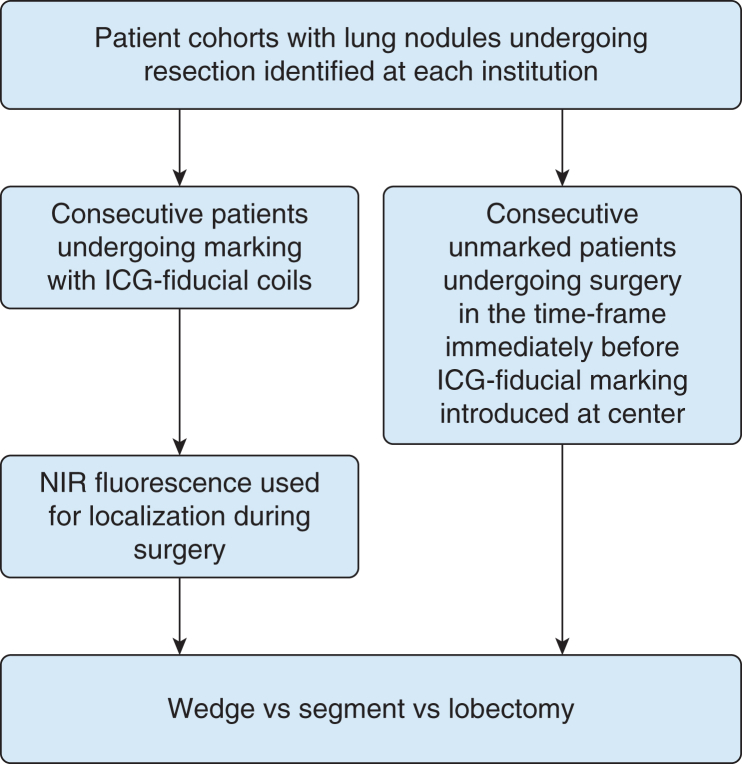
Flowchart representing patient selection for cohorts analyzed in the study. ICG = indocyanine green; NIR = near-infrared fluorescence.

Patients were evaluated at 4 centers ( El Camino Hospital/Palo Alto Medical Foundation (PAMF), Mountain View, California; Advocate Aurora Medical Center of Kenosha (AMCK), Kenosha, Wisconsin; Emory University, Atlanta, Georgia; and University of California, Davis, Davis, California) for newly found pulmonary nodules (Fig 1). At each center, all consecutive patients who underwent bronchoscopy with ICG-F before surgical resection were included in the ICG-F marked group. The decision to undergo ICG-F was at the discretion of the local medical team. Matching numbers of consecutive patients, stratified by each site, who had undergone robotic surgical resection without marking in the time period immediately prior were selected. This group comprised the unmarked group and consisted of patients who could not undergo ICG-F because this technique was not yet available at the institution.

Demographic data, nodule characteristics, procedural and surgical information, and final pathology results were obtained. The primary end point was the mean weight and size, measured in 3 dimensions of the resected lung specimens in each group. Secondary end points included incidence of complications from the bronchoscopy, including pneumothorax or bleeding. The study was approved by the institutional review boards of PAMF (#1014791-6), Advocate Aurora Research Institute (RAPR #378), Emory University (#IRB00046825), and University of California, Davis (#2136734-1).

### Procedure Description

Patients underwent ssRANB using the Ion Endoluminal System (Intuitive) with cone beam CT scan for secondary confirmation using either a fixed, ceiling mounted Artis Zee platform (Siemens Medical Solutions) (PAMF, n = 7), the mobile Cios Spin (Siemens Medical Solutions) (AMCK, n = 35; University of California, Davis, n = 2), or 2-dimensional fluoroscopy for guidance (Emory University, n = 10; University of California, Davis, n = 3). Lesions were either biopsied (n = 50) for a tissue diagnosis before marking (with on-site intraprocedural cytopathology available) or marking was performed immediately after navigation to the target was complete (n = 7) when there was a high clinical suspicion for malignancy based on serial imaging showing growth of the nodule.

ICG-F marking was performed throughout all institutions in the same manner as previously described^20^ (Videos 1-3). A total of 0.2 to 0.5 mL of a prediluted mixture of ICG (25 mg of ICG dye mixed with 10 mL of sterile water resulting in a 2.5 mg/mL ICG dye concentration; NDC-70100-424-02; HUB Pharmaceuticals) was drawn through a standard 1-mL tuberculin syringe with a Luer lock and injected into a 7 × 3 mm in tapering diameter from widest to narrowest × 8 cm in length Cook Tornado coil cartridge sheath (Cook Medical LLC). Ten minutes was chosen to prime the sheath of the catheter to allow the ICG solution to reach the fibers of the coil and soak the synthetic fibers of the coil within it with the ICG dye. The ICG-F was backloaded into an unmodified superDimension delivery catheter, an additional 0.2 to 0.5 mL of ICG dye was instilled within the coil sheath for further priming, and then the ICG-F was deployed by the included guidewire under fluoroscopic guidance (superDimension Marker Delivery Kit; Medtronic).

**Figure 2:**
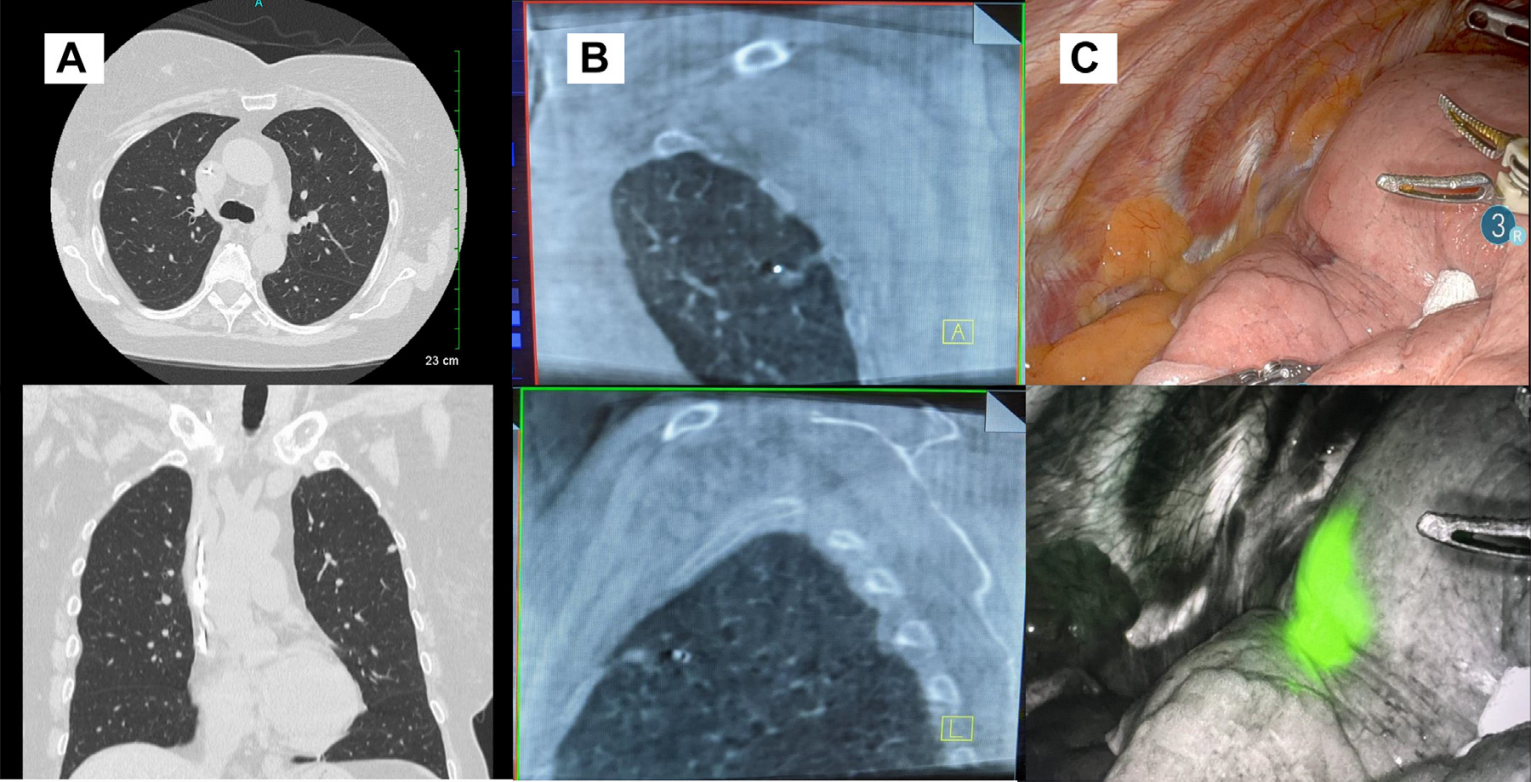
A-C, Representative imaging from indocyanine green-soaked fiducial marker (ICG-F) placement bronchoscopic procedure followed by robotic resection. A, Chest CT scan showing a 7-mm left upper lobe peripheral pulmonary lesion (PPL) in a patient with breast cancer and concern for metastatic disease. B, Cross-sectional imaging after ICG-F placement near the PPL via shape-sensing robotic-assisted bronchoscopy. C, Images from surgical resection showing absence of any visible indications of the PPL, lack of dye dispersion, and appropriate illumination of the target with the ICG-F marking when near-infrared imaging is used.

After lesion marking was complete, the patients were transported to a nearby operating room for robotic wedge resection (da Vinci; Intuitive) (8 total; PAMF, n = 5; University of California, Davis, n = 3) or transferred to recovery before discharge home for the surgery to occur at a later date (46 total) (Fig 2).

### Statistical Analysis

Numerical variables were summarized using descriptive statistics, including mean, SD, and range. Categorical variables were summarized using frequency and percentage distributions. To assess the association between categorical variables across the 2 groups (ICG-F marked and unmarked), χ^2^ tests and, where applicable, Fisher exact tests were used. When comparing the means of numerical variables between the 2 groups, a 2-tailed independent *t* test was used. A significance level (alpha) of .05 was applied across all statistical comparisons. This threshold was used to determine whether observed differences were statistically significant. All statistical analyses were performed using SAS version 9.4 (SAS Institute).

## Results

No differences were seen in age, race/ethnicity, sex, smoking status, pulmonary function tests values, or PET avidity of the lung nodules in patients between the 2 groups (Table 1). In both the ICG-F marked and unmarked groups, 3 patients each had 2 lung nodules and multiple patients had a history of prior malignancies (ICG-F marked: n = 16 [30%]; unmarked: n = 22 [37%]).

**Table 1:** Patient Characteristics

Characteristic	ICG-F Marked (n = 54)^a^	Unmarked (n = 60)^b^	*P* Value
Age, y	66 [12] (35-89)	67 [10] (36-90)	.7287
Race/ethnicity^c^			
White	38 (79)	48 (80)	.4254
Black	9 (19)	8 (13)	...
Other^d^	1 (2)	4 (7)	...
Sex			
Female	31 (57)	35 (58)	.9204
Male	23 (43)	25 (42)	...
Smoking status^c^			
Active	7 (14)	12 (20)	.4685
Never	9 (19)	15 (25)	...
Previous	32 (67)	33 (55)	...
PFT^e^			
FVC % predicted	87.42 [17.70]	94.22 [14.91]	.0527
FEV_1_ % predicted	73.42 [21.77]	81.80 [20.63]	.0587
FEV_1_/FVC	77.95 [17.38]	80.87 [16.23]	.4152
TLC % predicted	101.80 [18.94]	106.8 [18.62]	.2141
Dlco % predicted	76.12 [23.24]	75.86 [17.34]	.9527
PET scan^f^			
PET-avid	31 (69)	45 (83)	.0902
Non-PET-avid	14 (31)	9 (17)	...

Data are expressed as mean [SD] (range), mean [SD], No. (%), or as otherwise indicated. Dlco = diffusing capacity for carbon monoxide; ICG-F = indocyanine green-soaked fiducial marker; PFT = pulmonary function test; TLC = total lung capacity.

a Patients: n=35 Advocate Aurora Medical Center, Kenosha, n=8 Emory University, n=5 University of California, Davis, and n=6 Palo Alto Medical Foundation.

b Patients n=35 Advocate Aurora Medical Center, Kenosha, n=10 Emory University, n=5 University of California, Davis, and n=10 Palo Alto Medical Foundation.

c Missing: n = 6.

d Other = ICG-F marked: Asian (n = 1); unmarked: Asian (n = 2), Hispanic (n = 1), and unknown (n = 1).

e Missing: ICG-F marked: n = 11 for all; unmarked: FVC (n = 14), FEV_1_ (n = 9), FEV_1_/FVC (n = 14), TLC (n = 17), and Dlco (n = 10).

f Missing: n = 15.

Average lesion size was significantly different between the 2 groups in the axial (ICG-F marked: 14.39 ± 5.39 vs unmarked: 20.31 ± 14.24 mm; *P* = .0036) and coronal (ICG-F marked: 12.66 ± 5.13 vs unmarked: 16.43 ± 10.51 mm; *P* = .0214) dimensions (Table 2). Similarly, the distribution of the overall size of the nodules (< 2 cm in all dimensions vs > 2 cm in any dimension) and the presence or absence of a radiographic bronchus sign was significantly different across the 2 groups. Average distance from the nodule to the pleura was not statistically significantly different between the groups.

**Table 2:** Nodule Characteristics

Nodule	ICG-F Marked (n = 57)^b^	Unmarked (n = 63)^c^	*P* Value
Size, mm^a^			
Axial diameter	14.39 [5.39]	20.31 [14.24]	**.0036**
Coronal diameter	12.66 [5.13]	16.43 [10.51]	**.0214**
Sagittal diameter	12.00 [5.02]	14.34 [9.69]	.1315
< 2 cm in all dimensions	47 (82)	40 (66)	**.0373**
> 2 cm in any dimension	10 (18)	21 (34)	...
Type^d^			
Solid	42 (74)	54 (87)	.1792
Mixed	9 (16)	5 (8)	...
Ground-glass	6 (10)	3 (5)	...
Location^e^			
Left lower lobe	9 (16)	14 (23)	.4306
Left upper lobe	8 (14)	15 (24)	...
Right lower lobe	11 (20)	11 (18)	...
Right middle lobe	5 (9)	5 (8)	...
Right upper lobe	23 (41)	17 (27)	...
Lung 1/3^f^			
Central lung 1/3	8 (15)	12 (20)	.3500
Middle lung 1/3	13 (22)	19 (31)	...
Outer lung 1/3	35 (63)	30 (49)	...
Distance to pleura, mm			
Axial	6.76 [7.86]	8.75 [9.80]	.217396
Coronal	6.71 [6.62]	8.90 [9.99]	.077905
Sagittal	6.84 [7.60]	9.30 [9.67]	.121603
Bronchus sign^g^			
Present	46 (81)	32 (63)	**.0375**
Absent	11 (19)	19 (47)	...

Data are expressed as mean [SD], No. (%), or as otherwise indicated. Bold indicates any p value <0.05. ICG-F = indocyanine green-soaked fiducial marker.

a Missing: ICG-marked: axial (n = 1), coronal (n = 7), and sagittal (n = 7); unmarked: axial (n = 3), coronal (n = 9), sagittal (13), and > vs < 2 cm (n = 2).

b Nodules: Advocate Aurora Medical Center, Kenosha, n = 35; Emory University, n = 10; University of California, Davis, n = 5; and Palo Alto Medical Foundation, n = 7.

c Nodules: Advocate Aurora Medical Center, Kenosha, n = 35; Emory University, n = 12; University of California, Davis, n = 6; and Palo Alto Medical Foundation, n = 10.

d Missing: unmarked (n = 1).

e Missing: ICG-F marked (n = 1); unmarked (n = 1).

f Missing: ICG-F marked (n = 1); unmarked (n = 2).

g Missing: unmarked (n = 12).

**Figure 3:**
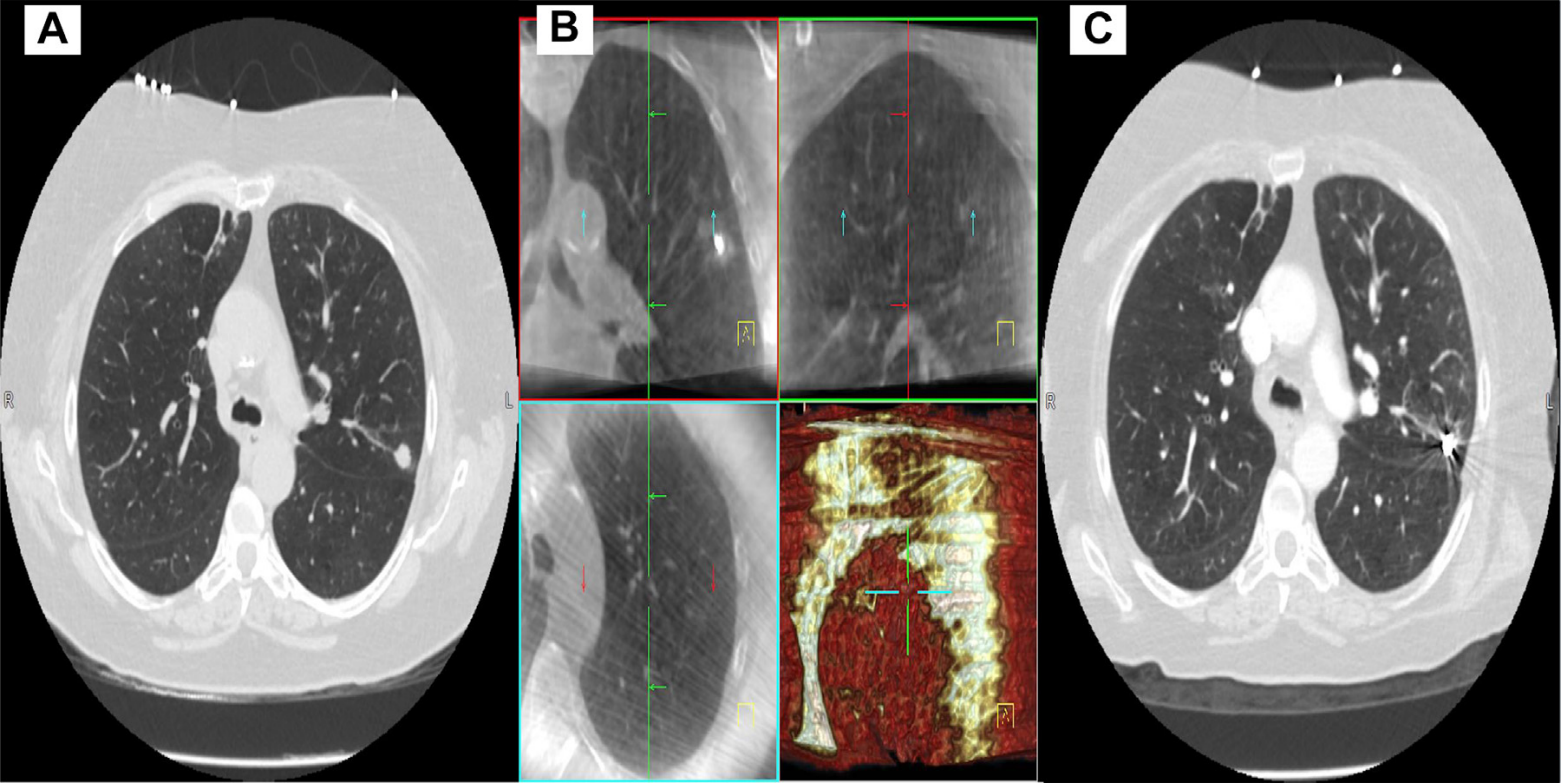
A-C, Representative imaging showing stability of indocyanine green-soaked fiducial marker (ICG-F) after placement during shape-sensing robotic-assisted bronchoscopy (ssRAB). A, Chest CT scan showing a left upper lobe peripheral pulmonary lesion. B, Cross-sectional imaging after ICG-F placement near the PPL via ssRAB. C, Stability of ICG-F after repeat chest CT 6 mo after ICG-F placement.

Out of 57 nodules in the ICG-F marked group, 7 were not sampled during bronchoscopy. Of the remaining 50 biopsied nodules, a diagnosis was confirmed in 78% (39 of 50). After bronchoscopy with ICG-F placement (n = 57), 1 patient with squamous cell carcinoma underwent radiation therapy instead of surgery, 1 patient was treated for infection due to positive cultures for non-TB mycobacteria, and 1 patient with squamous cell carcinoma declined further treatment. Follow-up imaging at least 6 months after ICG-F placement showed no change in the location of the markers (Fig 3). No pneumothorax or bleeding that required additional interventions other than standard airway suctioning occurred.

Patients in the ICG-F marked group underwent surgery either the same day (n = 8) or up to 13 days later (n = 46) (mean, 5.4 ± 3.1 days; range, 2-13 days; median, 5 days). Using a near-infrared illumination system (Firefly Fluorescence Imaging system) with the robotic system, the previously dyed nodules in the ICG-F marked group were all appropriately seen as a neon green luminescent target on the pleural surface.

In the ICG-F marked group, the weight of all the resected specimens (58 ± 77 g) was significantly decreased compared with all in the unmarked group (145 ± 80 g; *P* < .001). In subgroup analysis, a significant difference in weight was also seen in the segmentectomy group (ICG-F marked: 24 ± 29 vs unmarked: 162 ± 92 g; *P* < .001) and wedge resection group (ICG-F marked: 12 ± 16 vs unmarked: 23 ± 17 g; *P* = .0500) (Table 3). Similar results were seen when comparing the size of all the specimens in all measured dimensions (ICG-F marked: 9.07 ± 6.0 × 4.73 ± 3.6 × 2.42 ± 1.23 vs unmarked: 14.63 ± 6.08 × 8.70 ± 4.36 × 4.08 ± 1.94; *P* < .001 for all) and in the lobectomy and segmentectomy groups.

**Table 3:** Surgical Resection Results

Total	ICG-F Marked (n = 54)	Unmarked (n = 63)	*P* Value
Lung resection weight, g^a^	58 [77]	145 [80]	**< .001**
Lung resection size, mm			
First dimension	9.07 [6.0]	14.63 [6.08]	**< .001**
Second dimension	4.73 [3.6]	8.70 [4.36]	**< .001**
Third dimension^b^	2.42 [1.23]	4.08 [1.94]	**< .001**

Lobectomy	ICG-F Marked (n = 16)	Unmarked (n = 35)	*P* Value
Lung resection weight, g	153 [71]	177 [45]	.2160
Lung resection size, mm			
First dimension	14.24 [6.34]	18.31 [4.55]	**.0120**
Second dimension	8.58 [4.0]	11.20 [3.40]	**.0200**
Third dimension	3.59 [0.93]	5.09 [1.76]	**.0030**


Segmentectomy	ICG-F Marked (n = 11)	Unmarked (n = 11)	*P* Value
Lung resection weight, g^c^	24 [29]	162 [92]	**< .001**
Lung resection size, mm			
First dimension	7.47 [6.95]	14.21 [3.23]	**.0090**
Second dimension	3.66 [2.44]	9.01 [2.39]	**< .001**
Third dimension	2.34 [1.62]	3.76 [0.87]	**.0180**


Wedge Resection	ICG-F Marked (n = 27)	Unmarked (n = 17)	*P* Value
Lung resection weight, g^d^	12 [16]	23 [17]	**.0500**
Lung resection size, mm			
First dimension	6.49 [2.48]	7.34 [2.57]	.2800
Second dimension	2.76 [1.22]	3.34 [1.13]	.1220
Third dimension^b^	1.76 [0.63]	2.09 [1.04]	.2700

Data are expressed as mean [SD] or as otherwise indicated. Bold indicates any p value <0.05. ICG-F = indocyanine green-soaked fiducial marker.

a Missing: ICG-F marked (n = 3); unmarked (n = 6).

b Missing: ICG-F unmarked (n = 1).

c Missing: ICG-F marked (n = 1).

d Missing: ICG-F marked (n = 2); unmarked (n = 6).

During surgical resection, 4 patients in the unmarked group underwent an initial wedge resection and then completion lobectomy as planned due to anatomic location of the lesion and diagnosis obtained on initial frozen section. One patient in the unmarked group underwent initial wedge resection followed by conversion to lobectomy due to inability to visualize the target nodule. This conversion did not occur in the ICG-F group. All resected surgical specimens contained the ICG-F marker. Margins in both groups were at least 1 cm for all resected specimens.

Final pathology revealed a nonmalignant diagnosis for 3 patients in both the ICG-F marked group (necrotizing granulomas alone: n = 1, with acid-fast bacilli: n = 1, or with histoplasmosis: n = 1) and unmarked group (hamartoma: n = 1, necrosis and fibrosis: n = 1, necrotizing granulomas: n = 1). Metastatic disease was found in 10 and 6 patients in the ICG-F marked and unmarked groups, respectively. Although surgical time was statistically increased in the ICG-F marked group (ICG-F marked: 165 ± 53 vs unmarked: 136 ± 43; *P* = .0021), there were no differences in time to chest tube removal or discharge between the groups (Table 4).

**Table 4:** Results of Surgical Resections

Surgery	ICG-F Marked (n = 54)	Unmarked (n = 63)	*P* Value
Time, min^a^	165 [53]	136 [43]	**.0021**
Final pathology results			
Malignant diagnosis	51 (94)	60 (95)	.2060
Nonmalignant diagnosis	3 (6)	3 (5)	...
Postoperative events			
Chest tube removal, d^b^	1.90 [1.68]	2.76 [3.10]	.0650
Discharge, d^b^	2.63 [1.65]	2.77 [1.68]	.6425

Data are expressed as mean [SD], No. (%), or as otherwise indicated. Bold indicates any p value <0.05. ICG-F = indocyanine green-soaked fiducial marker.

a Missing: ICG-F marked (n = 7); unmarked (n = 3).

b Missing: ICG-F marked (n = 3); unmarked (n = 1).

## Discussion

We present our initial multicenter experience using ICG-F to mark PPLs. This approach was technically successful with the ability to clearly identify all lesions with ICG-F marking and allowed resection up to 13 days after bronchoscopy. We found that using this novel approach for small PPLs facilitates lung sparing surgery of otherwise undetectable lesions immediately after bronchoscopic placement or up to 13 days later without an increase in duration of chest tube placement or hospital stay; however, there was a statistically significant increase in total surgery time.

For pulmonary nodules that are > 5 mm from the pleural surface and < 10 mm in size, the probability of failing to detect the nodule during a video-assisted thoracoscopic surgery approach is > 60%.^33^ Additionally, pure ground-glass opacities, which are being detected with increasing prevalence on outpatient CT scans,^34^ are challenging to palpate. When the surgeon cannot visualize or palpate the lesion, conversion from a minimally invasive approach to an open thoracotomy may be required. Although this approach ensures the diagnosis, it also leads to increased postsurgical pain and length of stay.^35^ Although conversion rates have decreased over time, a meta-analysis reported a 9.6% median conversion rate with most due to technical issues (eg, difficult lymph node dissection, tumor size and/or location, challenging patient anatomy, presence of adhesions).^36^ Thus, successful localization of PPLs before resection may facilitate a minimally invasive surgical approach, which is more germane in an era of increasing interest in the use of sublobar resections for early stage lung cancers or potentially metastatic lesions.

Initial results with ssRANB for diagnosing peripheral pulmonary nodules have shown increased sensitivity for malignancy,^27^, ^28^, ^29^, ^30^, ^31^ including for lesions < 2 cm in diameter.^28^, ^29^, ^30^, ^31^ As such, we used ssRANB with cone beam CT scan or intraprocedural 3-dimensional mobile fluoroscopy for secondary confirmation to guide lesion marking. Although mean lesion size in the ICG-F marked group was significantly smaller, both groups contained a similar number of PPLs in the outer third of the lung and in their radiographic bronchus sign distribution (Table 2). Although some of these characteristics are usually associated with poorer diagnostic yields^37^, ^38^, ^39^ and may have impacted our results because only 78% (39 of 50) of the biopsied lesions had a confirmed bronchoscopic diagnosis, accurately targeting a nodule or another area (eg, pleural surface for dye, distal or proximal location for coil placement) for marking before surgical resection may offer a way to overcome this challenge. After bronchoscopy was completed, all ICG-F marked lesions were able to be clearly visualized with the near-infrared illumination system without broad dye dispersion. Nonpalpable lesions (eg, ground-glass and subsolid lesions) (n = 15, 26%) were marked with similar efficacy to solid nodules (n = 42, 74%) (Table 2). Additionally, metastatic lesions were also readily marked (n = 10, 18%). These scenarios speak to the broad applicability of this technique in various surgical situations, including lung sparing surgeries in which a wedge resection or segmentectomy may be preferred rather than an upfront lobectomy. In contrast, other scenarios exist in which marking may not be indicated (eg, more central lesions which would require a large amount of tissue removal to ensure clear margins; those cases in which an empirical lobectomy, large wedge resection, or anatomic resection are indicated). Future studies will thus be important to determine what patients may optimally benefit from a marking technique.

The use of new technologies also presents the possibility of new challenges. Surgical time was significantly increased by an additional 29 minutes in the ICG-F marked group (Table 4). The reason for this finding is unclear at this point, but may be attributed to the initial subjective assessment that the ICG-F marked nodules were more difficult to locate, which is supported by their smaller size in comparison with the unmarked lesions, thus necessitating them to be marked for localization. This increased surgical time could also be attributed to the need to meticulously dissect complex, deeper anatomic structures (eg, bronchovascular planes, lymphatic tissues) while performing segmentectomies and lobectomies for marked PPLs compared with taking a more generous piece of unmarked tissue.^40^ This hypothesis may be supported by the smaller size and weight of the marked surgical specimens in our resected segmentectomy and wedge resection subgroups (Table 3). Although we do note the lesions in the ICG-F marked group were significantly smaller (Table 2), thus potentially facilitating resection of less tissue, the limited sample size makes further assessment of this finding challenging. It is also possible that because more bronchoscopic marking procedures are performed at our institutions and others, the mean surgical time will decrease as seen in other studies.^41^^,^^42^ Reassuringly, no differences in clinical end points (eg, chest tube duration, length of stay) were found between the groups. Additionally, no patients in the ICG-F marked group required conversion to lobectomy to ensure that the lesion was removed, pointing to the value in accurately marking otherwise obscure lesions that may not be easily found and allowing for this to occur multiple days before surgery. Although our study was not designed to assess this difference in surgical time, future comparisons will need to be performed to determine if this increase persists and if so, elucidate possible explanations for it.

Although there appears to be value in this procedure to facilitate lung sparing surgery, dye and fiducial marking for PPLs are underused. In a prospective study of 1,215 patients undergoing electromagnetic navigation-guided bronchoscopy in the United States, 258 patients had fiducial markers placed,^9^ but only 23 patients had a dye marking procedure.^12^ However, the cohort had > 60% of its lesions located in the outer third of the lung with radiographic evidence of a limited disease and 6.3% of the lesions were classified as ground-glass opacity.^14^ It is unclear why lung nodule localization is not commonly performed. Potential barriers include access to the procedure/proceduralist, logistical challenges within institutions, and reliance on an open surgical approach.

Our initial findings showed that ICG-Fs retain their fluorescence-emitting abilities for at least 9 days after implantation at the time of surgery.^20^ Our current study increases that time to at least 13 days, thus allowing for even further time between bronchoscopic procedure and surgical resection. This approach may facilitate surgery for patients without ready access to thoracic surgeons, thus potentially solving 1 obstacle and allowing better outcomes at centers of excellence.^43^^,^^44^ Additionally, it may solve logistical challenges at institutions when trying to coordinate performing both marking and surgery under 1 anesthetic event. Studies identifying the patient groups that will derive maximal benefit from this approach are needed.

Our study does have its limitations. These include its design as a retrospective, comparative cohort study rather than a prospective, randomized controlled study and the potential for selection bias. Additionally, the absence of a formal agreed on selection process for patients between institutions may also lead to bias. Selection bias was attempted to be controlled by ensuring that all consecutive patients undergoing ICG-F marking from all centers were included and that consecutive unmarked patients were chosen from the time period immediately before a change in surgical approach, rather than by choosing patients at random. There were no significant differences in patient demographics between the 2 groups (Table 1), and the decreased size of the nodules in the ICG-F marked group (Table 2) would have led one to hypothesize that more tissue may need to be removed to ensure that a small, nonvisible, nonpalpable lesion would be appropriately removed. The study is also strengthened by its multicenter design and the elimination of any run-in period, speaking to its broad potential applicability to the evaluation, diagnosis, and management of small PPLs.

## Interpretation

Methods to improve PPL marking to facilitate surgical resection are imperative because more lesions will continue to be identified. From our experience, bronchoscopic ICG-F marking appears to be a safe and accurate approach to facilitate lung sparing surgery for otherwise undetectable lesions immediately after bronchoscopy or up to 13 days later. Although no increase in hospital length of stay or chest tube duration was identified, there was an increase in surgery duration. The ability for ICG-soaked coils to maintain their fluorescence for multiple days before surgery allows patients without direct access to expert thoracic surgical services to obtain care at centers far from home, with the possibility to improve outcomes. Further studies will need to be performed to determine the optimal patients that would benefit from this approach.

## Funding/Support

The authors have reported to *CHEST Pulmonary* that no funding was received for this study.

## Financial/Nonfinancial Disclosures

The authors have reported to *CHEST Pulmonary* the following: H. B. and G. K. have pending patent applications directed to methods of using dye-soaked fiducial coils for marking lesions in the lungs. None declared (B. S. B., E. M. C., M. D. P., W. B. T., A. N., W. S. J., O. V. K., K. D., C. T. P., L. A. G., D. A., M. L., E. A. Y., T. O., M. L., P. L. S.).

## Supplementary Data

Video 3

e-Online Data

## References

[1] Sung H., Ferlay J., Siegel R.L. (2021). Global Cancer Statistics 2020: GLOBOCAN estimates of incidence and mortality worldwide for 36 cancers in 185 countries. CA Cancer J Clin.

[2] Walters S., Maringe C., Coleman M.P. (2013). Lung cancer survival and stage at diagnosis in Australia, Canada, Denmark, Norway, Sweden and the UK: a population-based study, 2004-2007. Thorax.

[3] National Lung Screening Trial Research Team, Aberle D.R., Adams A.M. (2011). Reduced lung-cancer mortality with low-dose computed tomographic screening. N Engl J Med.

[4] de Koning H.J., van der Aalst C.M., de Jong P.A. (2020). Reduced lung-cancer mortality with volume CT screening in a randomized trial. N Engl J Med.

[5] Goldstraw P., Chansky K., Crowley J. (2016). The IASLC Lung Cancer Staging Project: proposals for revision of the TNM Stage Groupings in the Forthcoming (Eighth) Edition of the TNM Classification for Lung Cancer. J Thorac Oncol.

[6] Detterbeck F.C., Boffa D.J., Kim A.W., Tanoue L.T. (2017). The Eighth Edition Lung Cancer Stage Classification. Chest.

[7] Gould M.K., Tang T., Liu I.L. (2015). Recent trends in the identification of incidental pulmonary nodules. Am J Respir Crit Care Med.

[8] Gould M.K., Donington J., Lynch W.R. (2013). Evaluation of individuals with pulmonary nodules: when is it lung cancer? Diagnosis and management of lung cancer, 3rd ed: American College of Chest Physicians evidence-based clinical practice guidelines. Chest.

[9] Allen M.S., Deschamps C., Lee R.E., Trastek V.F., Daly R.C., Pairolero P.C. (1993). Video-assisted thoracoscopic stapled wedge excision for indeterminate pulmonary nodules. J Thorac Cardiovasc Surg.

[10] Murasugi M., Onuki T., Ikeda T., Kanzaki M., Nitta S. (2001). The role of video-assisted thoracoscopic surgery in the diagnosis of the small peripheral pulmonary nodule. Surg Endosc.

[11] Hirai S., Hamanaka Y., Mitsui N., Morifuji K., Uegami S. (2006). Role of video-assisted thoracic surgery for the diagnosis of indeterminate pulmonary nodule. Ann Thorac Cardiovasc Surg.

[12] Isaka T., Takahashi K., Maehara T., Masuda M. (2015). Intraoperative core needle biopsy under complete video-assisted thoracic surgery for indeterminate tumor of lung. Surg Endosc.

[13] Park C.H., Han K., Hur J. (2017). Comparative effectiveness and safety of preoperative lung localization for pulmonary nodules: a systematic review and meta-analysis. Chest.

[14] Altorki N., Wang X., Kozono D. (2023). Lobar or sublobar resection for peripheral stage IA non-small-cell lung cancer. N Engl J Med.

[15] Kamel M.K., Lee B., Harrison S.W., Port J.L., Altorki N.K., Stiles B.M. (2022). Sublobar resection is comparable to lobectomy for screen-detected lung cancer. J Thorac Cardiovasc Surg.

[16] Jiang S., Wang B., Zhang M. (2023). Quality of life after lung cancer surgery: sublobar resection versus lobectomy. BMC Surg.

[17] Saji H., Okada M., Tsuboi M. (2022). Segmentectomy versus lobectomy in small-sized peripheral non-small-cell lung cancer (JCOG0802/WJOG4607L): a multicentre, open-label, phase 3, randomised, controlled, non-inferiority trial. Lancet.

[18] Rusch V.W. (2023). Initiating the era of "precision" lung cancer surgery. N Engl J Med.

[19] Sakamoto T., Takada Y., Endoh M., Matsuoka H., Tsubota N. (2001). Bronchoscopic dye injection for localization of small pulmonary nodules in thoracoscopic surgery. Ann Thorac Surg.

[20] Anantham D., Feller-Kopman D., Shanmugham L.N. (2007). Electromagnetic navigation bronchoscopy-guided fiducial placement for robotic stereotactic radiosurgery of lung tumors: a feasibility study. Chest.

[21] Bowling M.R., Folch E.E., Khandhar S.J. (2019). Fiducial marker placement with electromagnetic navigation bronchoscopy: a subgroup analysis of the prospective, multicenter NAVIGATE study. Ther Adv Respir Dis.

[22] Bolton W.D., Cochran T., Ben-Or S. (2017). Electromagnetic navigational bronchoscopy reduces the time required for localization and resection of lung nodules. Innovations (Phila).

[23] Awais O., Reidy M.R., Mehta K. (2016). Electromagnetic navigation bronchoscopy-guided dye marking for thoracoscopic resection of pulmonary nodules. Ann Thorac Surg.

[24] Bowling M.R., Folch E.E., Khandhar S.J. (2019). Pleural dye marking of lung nodules by electromagnetic navigation bronchoscopy. Clin Respir J.

[25] Minnich D.J., Bryant A.S., Wei B. (2015). Retention rate of electromagnetic navigation bronchoscopic placed fiducial markers for lung radiosurgery. Ann Thorac Surg.

[26] Folch E.E., Pritchett M.A., Nead M.A. (2019). Electromagnetic navigation bronchoscopy for peripheral pulmonary lesions: one-year results of the prospective, multicenter NAVIGATE study. J Thorac Oncol.

[27] Fielding D.I.K., Bashirzadeh F., Son J.H. (2019). First human use of a new robotic-assisted fiber optic sensing navigation system for small peripheral pulmonary nodules. Respiration.

[28] Benn B.S., Romero A.O., Lum M., Krishna G. (2021). Robotic-assisted navigation bronchoscopy as a paradigm shift in peripheral lung access. Lung.

[29] Kalchiem-Dekel O., Connolly J.G., Lin I.H. (2022). Shape-sensing robotic-assisted bronchoscopy in the diagnosis of pulmonary parenchymal lesions. Chest.

[30] Styrvoky K., Schwalk A., Pham D. (2022). Shape-sensing robotic-assisted bronchoscopy with concurrent use of radial endobronchial ultrasound and cone beam computed tomography in the evaluation of pulmonary lesions. Lung.

[31] Low S.W., Lentz R.J., Chen H. (2023). Shape-sensing robotic-assisted bronchoscopy vs digital tomosynthesis-corrected electromagnetic navigation bronchoscopy: a comparative cohort study of diagnostic performance. Chest.

[32] Bawaadam H., Benn B.S., Colwell E.M. (2023). Lung nodule marking with ICG dye–soaked coil facilitates localization and delayed surgical resection. Ann Thorac Surg Short Rep.

[33] Suzuki K., Nagai K., Yoshida J. (1999). Video-assisted thoracoscopic surgery for small indeterminate pulmonary nodules: indications for preoperative marking. Chest.

[34] Woodard G.A., Udelsman B.V., Prince S.R. (2023). Brief report: increasing prevalence of ground-glass nodules and semisolid lung lesions on outpatient chest computed tomography scans. JTO Clin Res Rep.

[35] McKenna R.J., Houck W., Fuller C.B. (2006). Video-assisted thoracic surgery lobectomy: experience with 1,100 cases. Ann Thorac Surg.

[36] Power A.D., Merritt R.E., Abdel-Rasoul M., Moffatt-Bruce S.D., D'Souza D.M., Kneuertz P.J. (2021). Estimating the risk of conversion from video-assisted thoracoscopic lung surgery to thoracotomy-a systematic review and meta-analysis. J Thorac Dis.

[37] Gex G., Pralong J.A., Combescure C., Seijo L., Rochat T., Soccal P.M. (2014). Diagnostic yield and safety of electromagnetic navigation bronchoscopy for lung nodules: a systematic review and meta-analysis. Respiration.

[38] Wang Memoli J.S., Nietert P.J., Silvestri G.A. (2012). Meta-analysis of guided bronchoscopy for the evaluation of the pulmonary nodule. Chest.

[39] McGuire A.L., Myers R., Grant K., Lam S., Yee J. (2020). The diagnostic accuracy and sensitivity for malignancy of radial-endobronchial ultrasound and electromagnetic navigation bronchoscopy for sampling of peripheral pulmonary lesions: systematic review and meta-analysis. J Bronchology Interv Pulmonol.

[40] Tane S., Nishio W., Nishioka Y. (2019). Evaluation of the residual lung function after thoracoscopic segmentectomy compared with lobectomy. Ann Thorac Surg.

[41] Veronesi G., Galetta D., Maisonneuve P. (2010). Four-arm robotic lobectomy for the treatment of early-stage lung cancer. J Thorac Cardiovasc Surg.

[42] Cerfolio R.J., Bryant A.S., Skylizard L., Minnich D.J. (2011). Initial consecutive experience of completely portal robotic pulmonary resection with 4 arms. J Thorac Cardiovasc Surg.

[43] Goodney P.P., Lucas F.L., Stukel T.A., Birkmeyer J.D. (2005). Surgeon specialty and operative mortality with lung resection. Ann Surg.

[44] Al-Sahaf M., Lim E. (2015). The association between surgical volume, survival and quality of care. J Thorac Dis.

