# Supplementary figures and images for: Spot On: Indocyanine Green-Soaked Fiducial Markers for Lung Nodules Prior to Thoracic Surgery
Source: CHEST Pulm. 2024 Dec 25;3(1):100131. doi: 10.1016/j.chpulm.2024.100131 (PMC13418667; doi:10.1016/j.chpulm.2024.100131)

## Slide 1
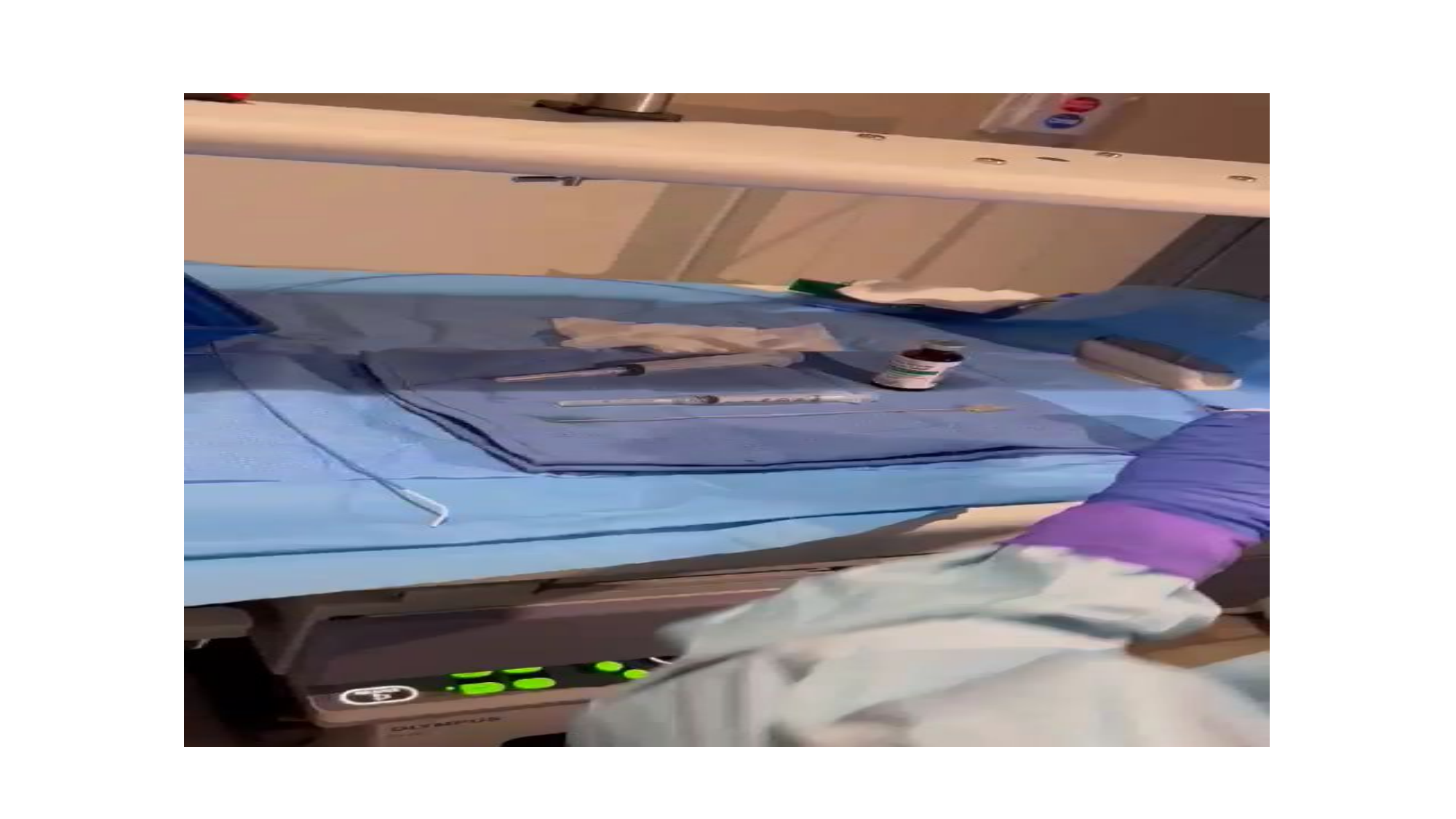

Supplement: Video 1 [file mmc1.pptx]
