# Supplementary material for: Spot On: Indocyanine Green-Soaked Fiducial Markers for Lung Nodules Prior to Thoracic Surgery
Source: CHEST Pulm. 2024 Dec 25;3(1):100131. doi: 10.1016/j.chpulm.2024.100131 (PMC13418667; doi:10.1016/j.chpulm.2024.100131)
Supplement: e-Online Data [file mmc4.docx]

Supplementary Videos Legend

Video 1: Priming the fiducial coil with indocyanine green dye while the coil is within the sheath

Video 2: Loading the indocyanine green dye soaked fiducial coil into the delivery catheter

Video 3: Deploying the indocyanine green dye soaked fiducial coil marker under fluoroscopic guidance
